# Responses to Song Playback Differ in Sleeping versus Anesthetized Songbirds

**DOI:** 10.1523/ENEURO.0015-22.2022

**Published:** 2022-05-23

**Authors:** Sarah W. Bottjer, Chloé Le Moing, Ellysia Li, Rachel Yuan

**Affiliations:** Section of Neurobiology, University of Southern California, Los Angeles, CA 90089

**Keywords:** anesthesia, sensory gating, sleep, songbird, spiking activity, vocal learning

## Abstract

Vocal learning in songbirds is mediated by a highly localized system of interconnected forebrain regions, including recurrent loops that traverse the cortex, basal ganglia, and thalamus. This brain-behavior system provides a powerful model for elucidating mechanisms of vocal learning, with implications for learning speech in human infants, as well as for advancing our understanding of skill learning in general. A long history of experiments in this area has tested neural responses to playback of different song stimuli in anesthetized birds at different stages of vocal development. These studies have demonstrated selectivity for different song types that provide neural signatures of learning. In contrast to the ease of obtaining responses to song playback in anesthetized birds, song-evoked responses in awake birds are greatly reduced or absent, indicating that behavioral state is an important determinant of neural responsivity. Song-evoked responses can be elicited during sleep as well as anesthesia, and the selectivity of responses to song playback in adult birds is highly similar between anesthetized and sleeping states, encouraging the idea that anesthesia and sleep are similar. In contrast to that idea, we report evidence that cortical responses to song playback in juvenile zebra finches (*Taeniopygia guttata*) differ greatly between sleep and urethane anesthesia. This finding indicates that behavioral states differ in sleep versus anesthesia and raises questions about relationships between developmental changes in sleep activity, selectivity for different song types, and the neural substrate for vocal learning.

## Significance Statement

Electrophysiological recordings of spiking activity in different taxa are heavily dependent on behavioral state. Neural activity patterns are frequently similar between sleeping and anesthetized animals, which has encouraged the idea that similar states characterize sleep and anesthesia. Based on comparisons across studies from our lab, we report that activity patterns are highly dissimilar between sleep and urethane anesthesia in a cortical region of juvenile songbirds. These data argue against the idea that similar behavioral states are achieved in sleep versus anesthesia.

## Introduction

Vocal learning in zebra finches serves as a powerful model for investigating mechanisms of motor skill learning during development (Doupe and Kuhl, 1999; Brainard and Doupe, 2013). Juvenile zebra finches learn the sounds used for vocal communication, and this type of skill learning, like other forms of goal-directed learning, is controlled by cortico-basal ganglia circuits (Yin and Knowlton, 2006; Graybiel, 2008; Redgrave et al., 2010; Turner and Desmurget, 2010; Cox and Witten, 2019). Similar to infants learning speech, juvenile songbirds memorize the vocal sounds of their adult tutor. They then progressively refine their own vocal behavior to imitate the tutor song (the goal behavior) during the sensorimotor stage of vocal learning. This process requires the evaluation of feedback of self-generated vocalizations against a neural representation of the goal tutor song to guide the gradual acquisition of an accurate imitation.

**Figure 1.**
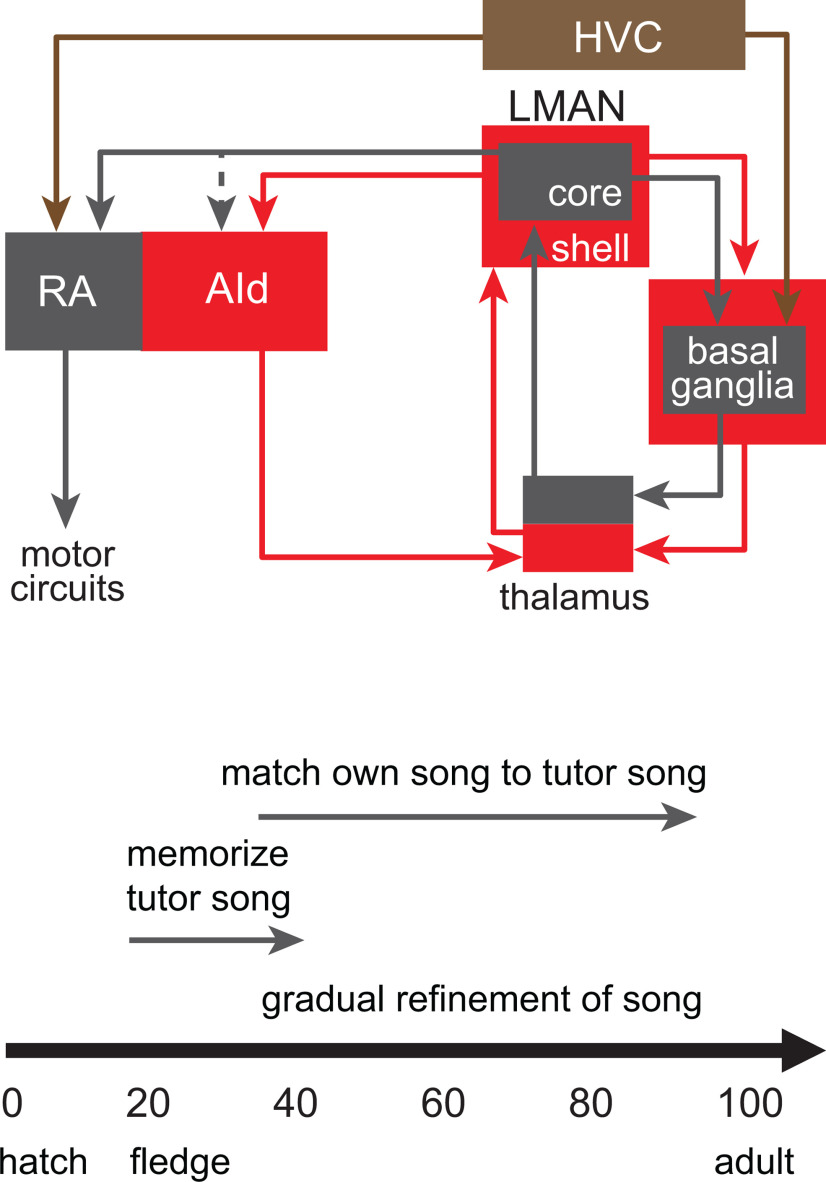
A simplified schematic of cortico-basal ganglia circuits that mediate vocal learning and a timeline of vocal development. Top, The cortical nucleus LMAN comprises CORE (gray) and SHELL (red) subregions which form parallel recurrent loops through the basal ganglia and dorsal thalamus. LMAN-SHELL also forms a trans-cortical loop via AId that converges with basal ganglia loops in the same dorsal thalamic zone. A transient projection from LMAN-CORE to AId is present only in juvenile birds and creates a site of integration between CORE and SHELL pathways in AId during early sensorimotor learning (denoted by dotted line). The dorsal thalamic zone feeds back to LMAN and feeds forward to HVC via medial MAN (latter pathway not shown for clarity). A specific region of the basal ganglia known as area X is dedicated to functions for vocal learning and includes both striatal and pallidal cells. RA: robust nucleus of the arcopallium; AId: dorsal intermediate arcopallium; HVC: high vocal center; LMAN: lateral magnocellular nucleus of the anterior nidopallium. Bottom, Zebra finches fledge from the nest ∼20 dph and are still reliant on parents to feed and preen them; juvenile males memorize the song of their biological father in the period from ∼20 to 35 dph. They begin to produce their first song-related vocalizations (babbling) ∼35 dph, and gradually refine their vocal motor output until they achieve a stable imitation of their memorized tutor song ∼80–90 dph; they produce a highly stereotyped song throughout adulthood.

Neural control of vocal learning in juvenile zebra finches is vested in basal ganglia loops that emanate from the cortical nucleus LMAN (Fig. 1; Bottjer et al., 1984; Scharff and Nottebohm, 1991; Aronov et al., 2008). CORE and SHELL subregions of LMAN make parallel connections through the basal ganglia and thalamus (Johnson et al., 1995; Iyengar et al., 1999; Luo et al., 2001; Iyengar and Bottjer, 2002a; Bottjer, 2004; Gale et al., 2008; Person et al., 2008; Paterson and Bottjer, 2017). The CORE pathway mediates vocal motor production in juvenile songbirds (Bottjer et al., 1984; Scharff and Nottebohm, 1991; Aronov et al., 2008; Elliott et al., 2014; Kojima et al., 2018) and is functionally similar to sensorimotor cortico-basal ganglia loops in mammals that contribute to learning and performance (Alexander and Crutcher, 1990; Graybiel, 2008; Yin et al., 2009; Ashby et al., 2010; Redgrave et al., 2010; Thorn et al., 2010; Gremel and Costa, 2013; Kupferschmidt et al., 2017). In contrast, the SHELL pathway is involved in evaluating sensorimotor performance and is functionally similar to associative-limbic loops that traverse the basal ganglia; lesions in the SHELL pathway of juvenile birds impair the ability to imitate tutor song, but do not cause motor disruption of song production (Bottjer and Altenau, 2010). This disruption of learning but not motor performance suggests that SHELL circuitry helps to evaluate whether self-generated vocalizations match learned tutor sounds.

Studies of the mechanisms that underlie vocal learning in songbirds have a long and venerable history of examining neural responses to playback of different song types in anesthetized juvenile and adult birds (Margoliash, 1983, 1986; Volman, 1993; Lewicki and Konishi, 1995; Solis and Doupe, 1997, 1999, 2000; Adret et al., 2012). One quest in this area was to discover a population of neurons that encode the tutor song memorized by each juvenile bird. Achiro and Bottjer (2013) reported that the SHELL subregion of LMAN in juvenile anesthetized birds contains a large proportion of neurons (∼30%) that respond significantly only to playback of tutor song. This tutor-tuned population provides a target memory that is essential for matching self-generated utterances to the goal tutor song, and is present only during early stages of sensorimotor integration. The proportion of tutor-tuned neurons diminishes during development as the incidence of neurons that responds selectively to each bird’s own song increases, suggesting that tutor-tuned neurons are lost (Johnson and Bottjer, 1992, 1993, 1994) or re-tuned to provide a template of self-generated song (Volman, 1993; Zevin et al., 2004; Nick and Konishi, 2005b; Kojima and Doupe, 2007; Achiro and Bottjer, 2013). In accord with the latter idea, the emergence of selectivity for each bird’s own song is a ubiquitous signature of vocal learning across forebrain regions including cortex (HVC, LMAN, RA), basal ganglia, and thalamus (Margoliash, 1983, 1986; Margoliash and Konishi, 1985; Margoliash and Fortune, 1992; Volman, 1993; Doupe, 1997; Solis and Doupe, 1997; Person and Perkel, 2007).

Thus, selective neural responses to playback of songs in anesthetized birds has highlighted the power of such experiments for studying mechanisms of vocal learning. However, several studies have shown that behavioral state is an important determinant of neural responsivity to song playback. Song-evoked responses can be elicited in sleeping as well as anesthetized zebra finches, and responses to song playback in adult birds are highly similar between anesthetized and sleeping states (Dave et al., 1998; Dave and Margoliash, 2000; Nick and Konishi, 2001), encouraging the idea that anesthesia and sleep states are highly similar. In contrast, song-evoked responses are greatly diminished or absent in awake zebra finches (Schmidt and Konishi, 1998; Cardin and Schmidt, 2003; Rauske et al., 2003; Cardin and Schmidt, 2004a, b), which is reminiscent of the suppression of auditory responses to self-generated sounds in both vertebrate and invertebrate taxa (Suga and Shimozawa, 1974; Poulet and Hedwig, 2006, 2007; Eliades and Wang, 2008; Singla et al., 2017; see Discussion). Here, we report that responses to playback of different song types in both CORE and SHELL subregions of LMAN in sleeping juvenile birds are substantially different from those that we reported previously in urethane-anesthetized birds of the same age under identical experimental conditions (Achiro and Bottjer, 2013). This difference stands in marked contrast to reports of similar song-evoked responsivity in sleeping and anesthetized adult songbirds (Dave et al., 1998; Dave and Margoliash, 2000; Nick and Konishi, 2001), and is consistent with recent data showing that urethane anesthesia does not mimic sleep states (Mondino et al., 2021).

## Materials and Methods

### Subjects

All procedures were performed in accordance with the University of Southern California’s animal care committee’s regulations. Five juvenile male zebra finches (*Taeniopygia guttata*) were bred in our group aviaries and remained there with their natural parents until 33 d posthatch (dph). At that time, they and their father were removed from the main aviary and housed in an individual cage in the recording chamber to habituate them to the space. Experimental birds therefore received normal social-auditory experience and exposure to the tutor song (their father’s song; Böhner, 1983, 1990; Mann et al., 1991; Mann and Slater, 1995; Roper and Zann, 2006).

### Electrophysiology

At 39 dph birds were anesthetized with isoflurane (1.5–1.8% inhalation) and placed in a stereotaxic apparatus. An electrode assembly consisting of eight tungsten-wire stereotrodes affixed to a movable microdrive was attached to the skull using dental cement such that the stereotrodes were implanted ∼300 mm dorsal to LMAN CORE and SHELL. Each stereotrode was a twisted pair of polyester polyamide-imide overcoated tungsten wires (25 μm in diameter, California Fine Wire Company) routed through fused silica capillary tubing (200 μm in diameter). The assembly consisted of four posterior stereotrodes and four anterior stereotrodes; a silver wire, placed between the skull and skin, served as animal ground. Following surgery each bird was housed in a small individual cage in the recording chamber adjacent to the cage with the father; the father was removed 4–6 d later.

One to 2 d following surgery, the stereotrode assembly was connected to a recording headstage (HS-16, Neuralynx) with a flexible cable connected to a commutator (PSR, Neuralynx); 15 channels of neural data were amplified, band passed between 300 and 5000 Hz (two Lynx-8 amplifiers, Neuralynx), and digitized at 32 kHz using Spike2 software (Power 1401 data acquisition interface, Cambridge Electronic Design). Audio and video were recorded coincident with neural activity: vocalizations were recorded to the 16th channel using a lavalier microphone (Sanken COS-11D) mounted in the cage, and two USB-video cameras (30 FPS, ELP Day Night Vision, X000UPN1M5, HD 1080p) were placed on opposite sides of the cage to record video files aligned to the neural activity. Two consecutive 60-min recordings were made between ∼8 and 10 P.M. starting about 1 h after lights off. Stereotrodes were manually advanced with the microdrive on consecutive days in the afternoon. The range of ages when recordings were made from LMAN CORE and/or SHELL ranged from 43 to 53 with a mean of 48.5 dph.

All birds received playback of four different songs: the bird’s own song (OWN, recorded within 24 h before each recording), the bird’s tutor song (TUT), a juvenile conspecific song (JuvCon), and an adult conspecific song (AdlCon). The latter two songs served as control stimuli for OWN song and TUT song, respectively. JuvCon songs were age-matched to the age of the experimental bird’s OWN songs. The order of stimuli within a block of four songs was random without replacement, and the interstimulus interval was 30 ± 1 s. Each song type was played back ∼50 times at an amplitude of 56–59 dB, but only playbacks that occurred during sleeping periods were used for analysis (see below).

At the end of each experiment, birds were perfused (0.7% saline followed by 10% formalin), and brains were removed and postfixed before being cryo-protected (30% sucrose solution) and frozen-sectioned in the coronal plane (50 μm thick). Sections were Nissl stained with thionin to visualize stereotrode tracks and verify recording locations. The border between CORE and SHELL subregions of LMAN was distinguished based on the density of magnocellular somata, which is low in SHELL relative to CORE.

### Data analysis

A recording site was considered for analysis if it was confirmed histologically to be in either LMAN-CORE or LMAN-SHELL (excluding 50 μm on either side of the CORE/SHELL border). The evoked responses of LMAN neurons tend not to persist throughout song stimuli longer than 1 s, as reported previously (Doupe, 1997; Solis and Doupe, 1997, 1999; Kojima and Doupe, 2007; Achiro and Bottjer, 2013). Therefore, response strengths calculated for song stimuli longer than 1 s underestimate the actual response by averaging across both the early phasic response and the period of decreased response. To correct for this stimulus duration bias (e.g., longer songs underestimate true response strengths), all analyses were performed using neural data collected during the first second of song playback.

Periods of sleep were scored manually by two independent observers; as a conservative estimate, only periods ranked as sleep by both observers were used for analysis. Careful examination of the video files was used to mark sleeping periods as those in which birds were completely quiescent, displaying a regular pattern of deep rhythmic breathing with their eyes closed for at least 10 s. Sleeping periods were terminated at least 2 s before onset of large movements (e.g., wing movements) or eye-opening. As indicated above, nonsleeping periods were eliminated such that only song playbacks that occurred during sleeping periods were included for analysis; the number of playbacks ranged from 16 to 48 (average 33 playbacks per song type in SHELL and 28 in CORE).

Movement artifact in multiunit neural recordings was correlated across recording channels and was eliminated or reduced using offline common average referencing: for each recording channel, the signal across the 14 remaining recording channels was averaged and subtracted from that channel to remove movement artifact (Ludwig et al., 2009). Noise was calculated as the standard deviation of the entire (2-h) voltage recording, and minimum signal-to-noise ratio was set as three times the standard deviation; this threshold was used for spike detection. Single units were sorted from multiunit data by first automatically clustering units with KlustaKwik (KD Harris, University College London). KlustaKwik clusters were manually inspected across 18 different waveform features and further refined using MClust 4.4 (A. D. Redish, University of Minnesota). Clusters were included for analysis if < 1% of spikes had an interspike interval (ISI) < 2 ms.

We determined whether each single unit was responsive to song playback by testing for a significant change in firing rate (excitation or suppression) between baseline and each song type (Wilcoxon signed-rank test, *p* < 0.05). Baseline periods were defined as 1-s periods immediately before stimulus playback, with the restriction that they must fall within sleep periods. For each song playback, the firing rates during the two closest baseline periods were averaged to generate a corresponding baseline value. To compare differences in firing rates across neurons, standardized response strengths (RS) were calculated as:

$$\text{standardized response strength }\left(\text{RS}\right)\;=\frac{\bar{S}\;-\;\bar{B}}{\sqrt{Var\left(S\right)\;+\;Var\left(B\right)\;-\;2\text{ * }Covar\left(S,B\right)}}.$$

Where *S* is the firing rate (spikes/s) during stimulus, and *B* is the firing rate during baseline, such that a positive value indicates an increased rate to a stimulus (excitation) and a negative value indicates a decreased rate (suppression). We refer to the standardized response strength as “response strength” (RS) throughout the text.

We report RS values in three different ways. (1) In order to assess response strengths across both excited and suppressed responses, we report absolute values of RS. (2) We report response strengths for excited and suppressed responses separately. In this measure, we calculated firing rates for all excited responses (all RS values greater than zero) and all suppressed responses (all RS values less than zero), and omitted RS values of zero (indicating the same firing rate during both stimulus and baseline) since they are neither excited nor suppressed. (3) We report significant excited and suppressed responses, i.e., including only RS values in which the firing rate during a stimulus was significantly different from baseline.

**Table 1:** Proportions of significant responses by song type (63 significant song-evoked responses produced by 49 SHELL neurons, 44 significant song-evoked responses produced by 35 CORE neurons)

	AdlCon	JuvCon	TUT	OWN
SHELL				
All responses	0.22 (14/63)	0.33 (21/63)	0.11 (7/63)	0.33 (21/63)
Excited	0.10 (6/63)	0.03 (2/63)	0.03 (2/63)	0.05 (3/63)
Suppressed	0.13 (8/63)	0.30 (19/63)	0.08 (5/63)	0.29 (18/63)
CORE				
All responses	0.20 (9/44)	0.43 (19/44)	0.11 (5/44)	0.25 (11/44)
Excited	0.02 (1/44)	0.14 (6/44)	0.00 (0/44)	0.05 (2/44)
Suppressed	0.18 (8/44)	0.30 (13/44)	0.11 (5/44)	0.21 (9/44)

To measure song selectivity for each song type for each cell, a difference score between response strength values was calculated for “SongA” as follows: Song A_ΔRS_ = RS_SongA_ – RS_SongB_. For example, positive scores obtained by subtracting response strengths to OWN, AdlCon, and TUT (“SongB” comparison songs) from JuvCon (“SongA” reference song) would indicate selectivity for JuvCon. This measure is similar to the psychometric discriminability index d’ except that responses are standardized before being subtracted, as opposed to subtracting response strengths and then dividing by the standard deviations as in d’ (see Achiro and Bottjer, 2013). This approach corrects for potential limitations of d’ scores, which are sensitive to response variability as well as response strength (Coleman and Mooney, 2004). Difference scores for song-suppressed responses were reversed in sign so that in all cases (both excited and suppressed responses) a positive difference score indicates a preference for the reference song over the comparison song while a negative difference score indicates a preference for the comparison song.

The conditions of both collecting and analyzing the data reported here are identical to the procedures used by Achiro and Bottjer (2013), including age and breeding population of birds, equipment and experimental setup, and scripts for analysis.

### Statistics

We used nonparametric statistics because of non-normal distributions of data, differing numbers of significant responses between song types, and differing numbers of neurons between CORE and SHELL regions. Differences in proportions were tested using χ^2^ or Fisher’s exact tests, and differences in distributions were tested with Kolmogorov–Smirnov Z tests. Friedman tests were used to evaluate differences in RS between song types (as a repeated measure) within CORE and SHELL regions, whereas Kruskal–Wallis tests were used to evaluate differences between song types for significant excitatory and suppressed responses within each region (because of differing number of responses). Wilcoxon signed-rank tests were used to assess individual differences between song types for all responses, and for comparing firing rates and burst fractions during sleep versus nonsleep periods; Benjamini–Hochberg corrections were used for multiple comparisons (Benjamini and Hochberg, 1995). Mann–Whitney tests were used to assess individual differences between song types for excited versus suppressed responses, which were also corrected for multiple comparisons using Benjamini–Hochberg. All values are given as mean ± SEM unless specified otherwise.

## Results

### Different song types elicited different proportions of responses in both CORE and SHELL regions of LMAN

**Figure 2.**
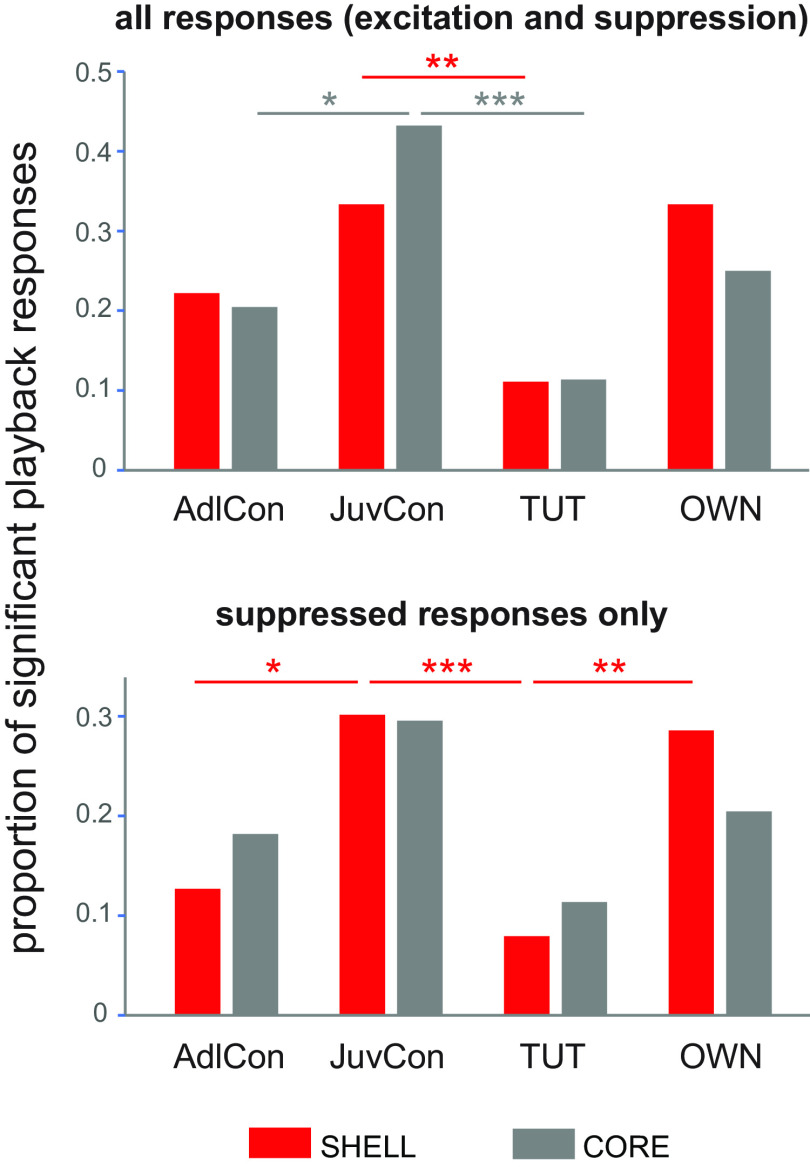
Proportion of significant responses to each song stimulus in CORE (gray) versus SHELL (red) neurons. Top, Proportions of excited and suppressed responses to playback of each song type (see Table 1). *p = 0.04, **p = 0.01, ***p = 0.003. Bottom, Proportions of suppressed responses to each song type. *p = 0.03, **p = 0.01, ***p = 0.006. AdlCon, adult conspecific song; JuvCon, juvenile conspecific song; TUT, tutor song; OWN, bird’s own song. *n* = 44 responses in 35 CORE neurons; *n* = 63 responses in 49 SHELL neurons.

We recorded from CORE and SHELL subregions of LMAN in sleeping juvenile zebra finches (43–53 dph, mean = 48.5 dph). By this age juveniles have completed memorization of their tutor’s song and begun to practice their incipient song vocalizations. All neurons (*n* = 66, CORE; *n* = 104, SHELL) were tested with four different song types: each bird’s own song (OWN), each bird’s tutor song (TUT), an age-matched song from a juvenile conspecific (JuvCon) and an adult conspecific song (AdlCon). Approximately half of the neurons in both CORE and SHELL showed a significant change in firing rate to at least one of the song types presented (CORE: 0.53, 35/66; SHELL: 0.47, 49/104); thus, both regions showed similar levels of responsivity to song playback (χ^2^ = 0.57, *p* = 0.45). Proportions of significant playback responses varied by song type within both CORE and SHELL (CORE: χ^2^ = 13.8, *p* = 0.003; SHELL: χ^2^ = 12.6, *p* = 0.006; Fig. 2, top panel; Table 1). JuvCon song elicited the highest proportion of responses whereas TUT evoked the lowest. Individual comparisons showed that the incidence of evoked responses to JuvCon was higher than that to TUT (CORE: *p* = 0.003; SHELL: *p* = 0.01, Fisher’s exact test, Benjamini–Hochberg corrected). JuvCon song also elicited a higher proportion of responses in LMAN-CORE neurons compared with AdlCon song (*p* = 0.04); no other comparisons between JuvCon and other song types were significant across all responses (*p* > 0.09 or higher).

Although we found evidence of both excitation and suppression, the majority of cells within both CORE and SHELL showed only suppressed responses. Approximately 75% of cells in CORE and SHELL were suppressed by song playback, whereas relatively few cells responded with only excitation or a combination of excitation and suppression to different song types (Table 2). The dominance of suppressed responses was clear for all four song types, but was particularly pronounced for the two song types that elicited the highest percentage of responses, JuvCon and OWN (Table 1). We therefore examined the proportions of suppressed responses elicited by different song types (Fig. 2, bottom panel). In contrast to comparison across all playback responses (Fig. 2, top panel), the bottom panel of Figure 2 shows that only SHELL neurons showed differential suppression between song types (SHELL: χ^2^ = 16.0, *p* = 0.001; CORE: χ^2^ = 4.99, *p* = 0.173). Within SHELL neurons, JuvCon evoked a higher incidence of suppressed responses compared with both TUT and AdlCon, but not OWN (TUT: *p* = 0.006; AdlCon: *p* = 0.034, Fisher’s exact test, Benjamini–Hochberg corrected). OWN song also evoked a higher proportion of suppressed responses compared with TUT (TUT: *p* = 0.011; OWN vs AdlCon was marginally significant, *p* = 0.057; Fisher’s exact test, Benjamini–Hochberg corrected). Thus, the proportion of suppressed responses varied by song type in SHELL, but not CORE; within SHELL neurons both JuvCon and OWN elicited a high incidence of suppressed responses relative to AdlCon and (especially) TUT.

**Table 2:** Proportions of neurons by response type

SHELL (*n* = 49)	# cells	proportion	CORE (*n* = 35)	# cells	proportion
Excitation only	8	0.163		4	0.114
Suppression only	37	0.755		26	0.743
Both	4	0.082		5	0.143

**Figure 3.**
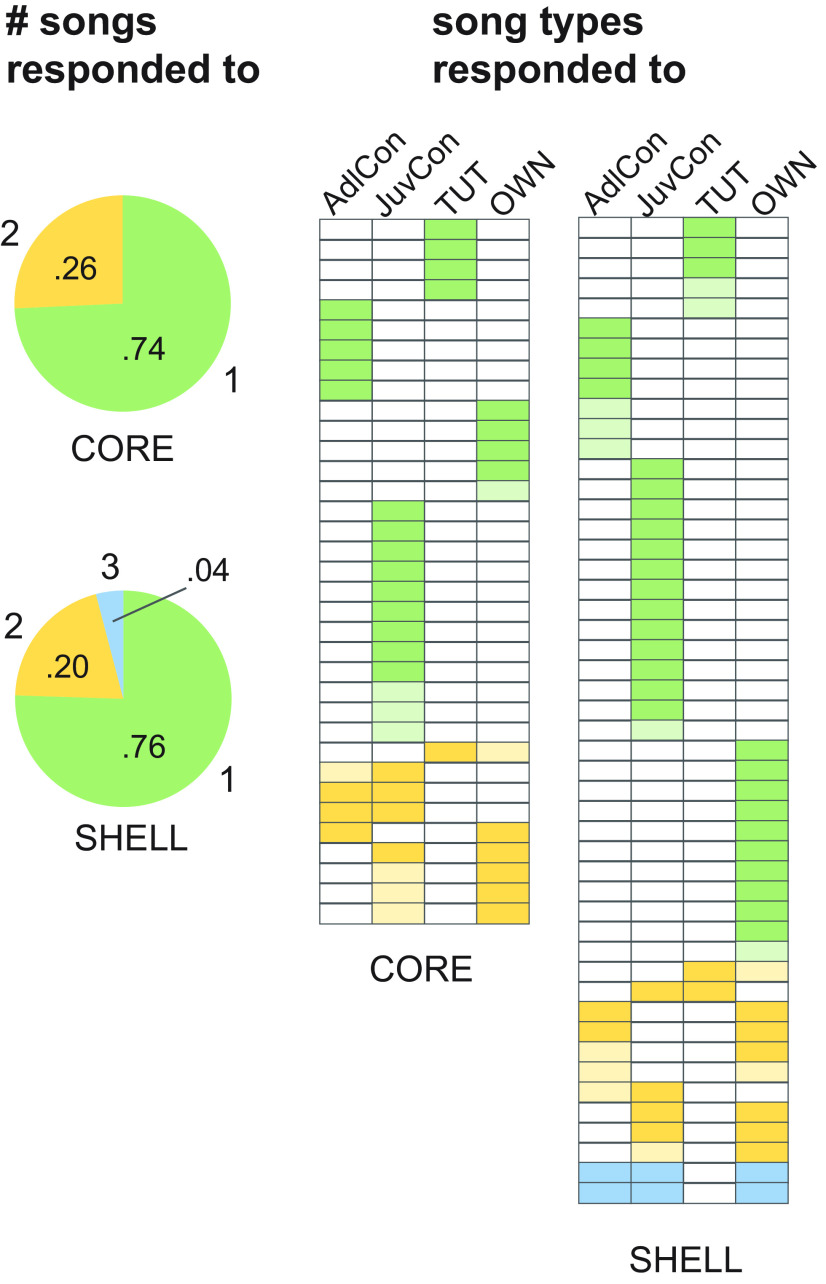
Single neurons were selectively tuned in both CORE and SHELL. Left, Proportions of neurons that responded to different numbers of songs out of the four song types played; most neurons (∼75%) responded to only one song stimulus in both CORE and SHELL (green), some neurons (20–26%) responded to two songs (yellow), and two neurons (4%) in SHELL responded to three songs. Right, Each row corresponds to one neuron, indicating the song stimuli to which each neuron responded (*n* = 44 responses in 35 CORE neurons; *n* = 63 responses in 49 SHELL neurons). Rows are ordered according to whether each neuron responded to one, two, or three songs (colors corresponding to those on the left). Columns depict responses to each song type, with darker shading indicating suppressed responses and lighter shading indicating excited responses; unshaded boxes depict nonsignificant responses.

Individual neurons were not broadly tuned: almost all neurons responded to either one or two of the four song types played; CORE neurons responded to 1.26 ± 0.07 different songs on average, whereas SHELL neurons responded to 1.29 ± 0.08. Figure 3, left, shows that ∼75% of neurons in both CORE and SHELL subregions responded to only one song type (green shading); the majority of the remaining cells responded to only two song types (yellow shading); no CORE neurons and only 4% of SHELL neurons responded to three songs (blue shading). Figure 3, right side, depicts the song types to which each individual neuron responded (dark shading, suppressed responses; light shading, excited responses), confirming that a low proportion of neurons in both CORE and SHELL responded to playback of TUT song, while relatively high proportions responded to both JuvCon and OWN songs.

To summarize these data based on proportions of song-evoked responses in sleeping juvenile birds during the period of sensorimotor integration: (1) neurons in both subregions of LMAN responded in a selective fashion to song stimuli; (2) all songs were more likely to elicit suppression of firing rates rather than excitation, especially JuvCon and OWN songs; (3) SHELL neurons showed a greater tendency toward suppressed responses to JuvCon and OWN songs compared with CORE neurons. Neurons at the population level evinced a preference for juvenile songs over adult songs, regardless of whether the juvenile song was self-generated (OWN) or produced by an age-matched conspecific bird (JuvCon).

This overall pattern of results contrasts markedly with that observed in our previous study in which birds of the same age were urethane-anesthetized rather than sleeping (Achiro and Bottjer, 2013). In that study, neurons in CORE were more likely to respond to playback compared with those in SHELL (0.89 vs 0.68), and neurons in both CORE and SHELL were much more likely to show excitation: CORE neurons never showed suppressed responses whereas ∼80% of responses in SHELL neurons were excitatory and ∼20% were suppressed. In addition, our prior work revealed a large proportion of SHELL neurons that exhibited a significant response only to TUT compared with those in CORE (0.28 vs 0.04), whereas a large proportion of CORE neurons responded to TUT plus other songs compared with SHELL neurons (0.43 vs 0.15; Achiro and Bottjer, 2013). Thus, the SHELL region of LMAN in anesthetized birds contains two distinct populations of neurons during early sensorimotor integration (45 dph): a larger one that responds only to the tutor song and a separate smaller population that responds only to the bird’s own song. In general, CORE neurons in anesthetized birds of this age are much more broadly tuned than SHELL neurons and show little evidence of selective responsivity to tutor song (see Achiro and Bottjer, 2013). The current data did not replicate any of these patterns in sleeping birds (see Discussion; Table 3).

**Table 3:** Comparison of current results with those by Achiro and Bottjer (2013)

	Anesthetized		Sleeping	
	CORE	SHELL	CORE	SHELL
% song-evoked neurons^*a*^	89	68	53	47
% suppressed neurons^*b*^	0	∼20	74	76
% TUT-only responsive neurons^*c*^	4	28	11	10
Selectivity score JuvCon vs TUT^*d*^	0.13	0.43	0.47	0.45
Selectivity score JuvCon vs OWN^*d*^	0.18	0.39	0.30	0.34

Anesthetized values are taken from Achiro and Bottjer (2013); sleeping values are taken from current study. Each measure in the table was significantly different between CORE and SHELL in anesthetized birds whereas none of the comparisons varied between CORE and SHELL in sleeping birds. The mean age of birds at which recordings were made by Achiro and Bottjer (2013) was 45.5 dph (range 43–47); the mean age of birds from recordings in the current study was 48.5 dph (range 43–53).

a percentage of neurons that responded to playback of at least one song.

b percentage of neurons that were suppressed by song playback (cells that showed suppression only are included for both studies).

c percentage of neurons that gave a significant response only to TUT and not to any other stimulus [out of five songs by Achiro and Bottjer (2013), out of four songs for the current study].

d Selectivity scores for JuvCon versus TUT, OWN refer to average difference scores between standardized response strengths (see Materials and Methods). Scores from present study are for suppressed responses among JuvCon-selective cells, while scores by Achiro and Bottjer (2013) are for cells for TUT-selective and OWN-selective cells, respectively (Achiro and Bottjer scores include excited responses for CORE and excited and suppressed responses for SHELL); we chose to present scores for JuvCon-responsive cells from this study since so few neurons responded to TUT (Table 1).

### Firing rates during sleeping versus nonsleeping periods

**Figure 4.**
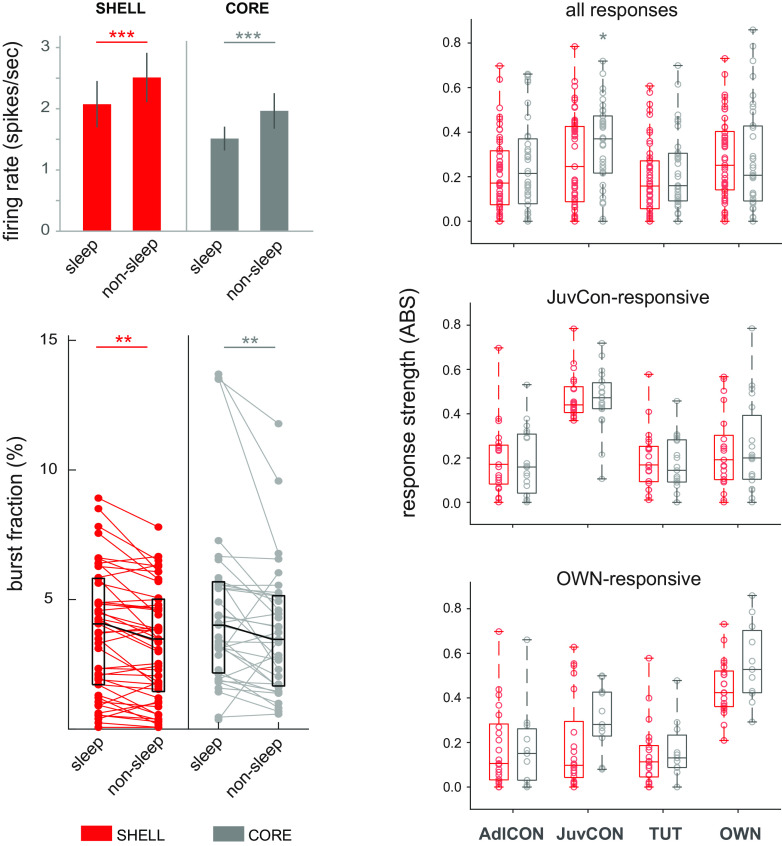
Firing rates and standardized response strengths in SHELL and CORE neurons. Left panel, Top shows spontaneous firing rates (averages ± SEM) during periods marked as sleeping versus nonsleeping; bottom shows burst fractions (percent of ISIs < 5 ms). One CORE neuron and two SHELL neurons were omitted from the graph of burst fractions since they were outliers (but were included in statistical analyses). **p < 0.005, ***p < 0.0001. Right panel, All graphs depict absolute values (ABS) of standardized response strengths as a function of song type. Top, Response strengths (including both excitatory and suppressed responses, both significant and nonsignificant) for all CORE and SHELL neurons (*n* = 35 CORE, *n* = 49 SHELL). * indicates main effect between songs in CORE, *p* = 0.039. Middle, Response strengths for the subset of CORE and SHELL neurons that showed a significant response to JuvCon song (*n* = 19 CORE, *n* = 21 SHELL). Bottom, Response strengths for the subset of CORE and SHELL neurons that showed a significant response to OWN song (*n* = 11 CORE, *n* = 21 SHELL). Box-and-whisker plots depict medians and first and third quartiles; whiskers in right panel indicate minimum and maximum values, and circles represent individual data points.

We are confident that we measured spiking responses to song playback during periods of sleep since the use of behavioral criteria has been shown to be highly reliable (Szymczak et al., 1996; Low et al., 2008). In addition, neural measures were consistent with our behavioral scoring: spontaneous firing rates (spikes/s) during the night were lower during periods marked as sleep compared with nonsleep (Wilcoxon signed-rank tests *p* < 0.0001 for both CORE and SHELL), and the percent of spikes that occurred in bursts (ISIs < 5 ms) was higher during sleep periods than during nonsleep periods (Wilcoxon signed-rank tests *p* < 0.005 for both CORE and SHELL; Fig. 4, left panel). This pattern is consistent with that observed in thalamocortical neurons in mammals, which fire at high regular rates during waking versus low rates interspersed with bursts during sleep or anesthesia (Steriade and Llinás, 1988; Swadlow and Gusev, 2001; Weyand et al., 2001). A similar pattern has been observed in adult songbirds: spontaneous bursting frequently occurs during sleep in neurons of the motor pathway (HVC and RA; Fig. 1), but not in awake nonsinging birds, and sleep bursts are dependent on bursting activity in the thalamic nucleus Uva (uvaeform nucleus; Yu and Margoliash, 1996; Dave et al., 1998; Hahnloser et al., 2002, 2008).

### Response strengths in subsets of CORE and SHELL neurons were selective for specific songs

Figure 4, right panel, shows absolute values of response strengths for all responses to each song type (including both excitatory and suppressed responses) for CORE and SHELL neurons. This measure revealed no difference in firing rates between songs in SHELL but a significant difference in CORE (Friedman test: CORE, *p* = 0.039; SHELL, *p* = 0.257); however, no individual comparisons were significant for CORE neurons despite the stronger response to JuvCon relative to other songs (*p* = 0.076 for JuvCon vs TUT, Wilcoxon signed-rank tests, Benjamini–Hochberg corrected). Given the relatively large proportion of significant responses to JuvCon song in both CORE and SHELL (Fig. 2), we compared absolute values of response strengths across song types for the subset of neurons that responded significantly to JuvCon (*n* = 19 CORE, *n* = 21 SHELL). Figure 4, right, middle, shows that this subpopulation in both CORE and SHELL exhibited a significantly higher firing rate to JuvCon compared with the other three song types (Friedman tests, *p* < 0.0001 in both CORE and SHELL; Wilcoxon signed-rank tests for JuvCon vs other song types always *p* < 0.004 or lower, Benjamini–Hochberg corrected). To determine whether this selective increase in firing rate was restricted to JuvCon-responsive neurons, we calculated firing rates for each subset of neurons that showed a significant response to the remaining three song types. A similar pattern was obtained for OWN-responsive, AdlCon-responsive, and TUT-responsive neurons, showing that single neurons that responded significantly to a given song type also showed a higher firing rate to that song type compared with other song stimuli. For example, OWN-responsive neurons in both CORE and SHELL had significantly higher response strengths to OWN compared with all other songs (Wilcoxon signed-rank tests for OWN vs other song types in SHELL always *p* < 0.001; in CORE always *p* < 0.005; Benjamini–Hochberg correction for multiple comparisons; Fig. 4, right, bottom). (We did not perform statistical tests for TUT-responsive or AdlCon-responsive neurons because of relatively low *n*s; see Table 1.)

**Figure 5.**
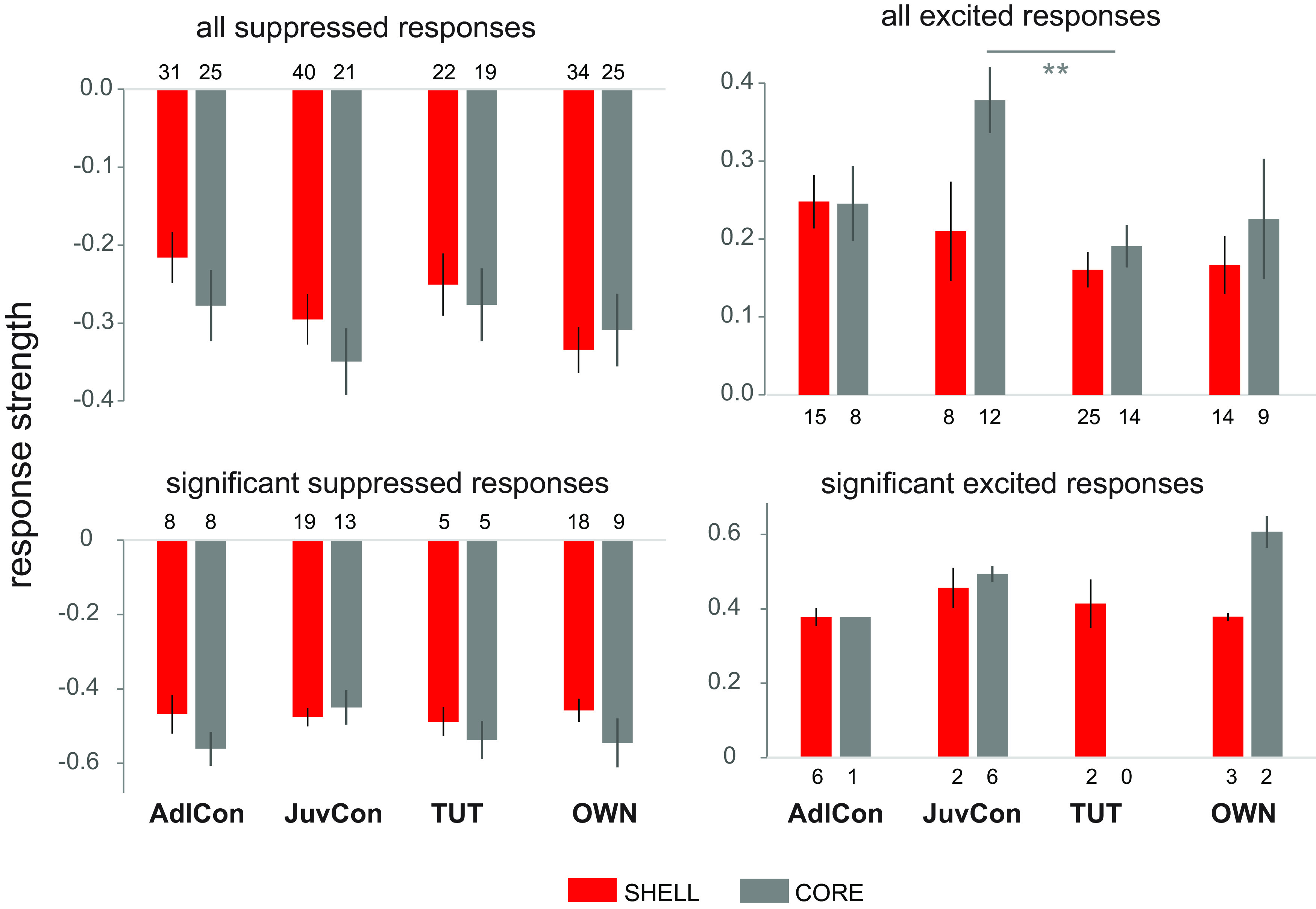
Average response strengths to each song stimulus for suppressed versus excited responses. Top panels, Suppressed responses including all response strengths less than zero (left) and excited responses including all response strengths greater than zero (right). Bottom panels, Significant responses (firing rates were significantly different from baseline), plotted as in top panels. Numbers just above/below each bar represent *n*s (*n*s for significant responses are also given in Table 1). Averages ± SEM.

Figure 5, top panels, presents all suppressed responses (less than zero) versus all excited responses (greater than zero). Interestingly, the trend toward stronger responses to JuvCon in CORE neurons shown in the top panel of Figure 4 was vested in excitatory responses: the firing rate to JuvCon in CORE neurons was greater compared with other songs for excited responses but not for suppressed responses (Kruskal–Wallis tests for CORE neurons: excitatory *p* = 0.024, suppressed *p* = 0.539; Mann–Whitney tests showed that individual comparisons were significant only for JuvCon vs TUT excited responses, *p* = 0.003, Benjamini–Hochberg correction). This pattern is consistent with that shown in Figure 2, in which differences in song responsivity were no longer significant in CORE neurons for suppressed-only responses. In contrast to CORE, suppressed, but not excited responses in SHELL neurons showed a significant overall difference in firing rates between song stimuli (Kruskal–Wallis tests for SHELL neurons: excitatory *p* = 0.228, suppressed *p* = 0.041), although no individual comparisons were significant (*p* > 0.17 in all cases, Benjamini–Hochberg correction for multiple comparisons). However, these differences in song-evoked firing rates were not evident when only significant responses were examined (Fig. 5, bottom panels)-statistical analyses performed on suppressed responses were not significant (Kruskal–Wallis: SHELL *p* = 0.97; CORE *p* = 0.51; excited responses were not assessed because of the small number of significant responses). The lack of any differences between significant responses to songs indicates that the enhanced firing rate seen in CORE neurons across excited responses (Fig. 5, top right panel) was because of responses that were stronger to JuvCon song but fell short of significance; when only significant excited responses were considered (Fig. 5, bottom right panel), this tendency was eliminated. Thus, significant suppression and excitation did not vary as a function of song type.

**Figure 6.**
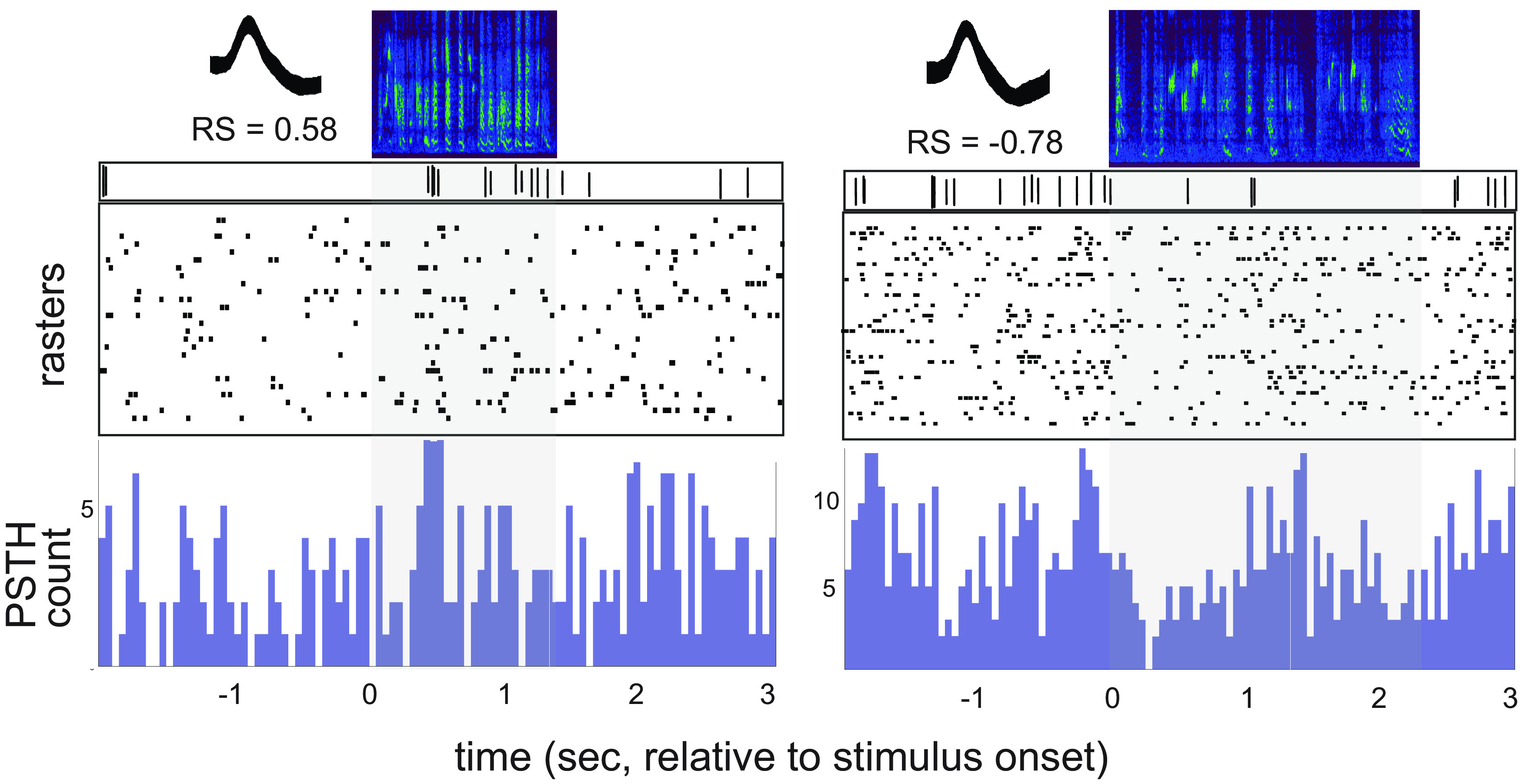
Two example single units during playback of JuvCon song. Units are from the same bird at 44 dph. Left, CORE neuron that showed excitation to JuvCon. Right, SHELL neuron that showed suppression to JuvCon. For each panel, top half shows song spectrograms and raw traces of single-unit activity; bottom half shows rasters and PSTHs. Overlaid waveforms shown in inset at top left; RS, mean response strength.

Figure 6 shows raster and poststimulus time histograms (PSTHs) for example neurons from LMAN in a bird that was 44 dph. The left panel shows a CORE neuron that showed excitation to playback of JuvCon song, and the right panel shows a SHELL neuron in which the firing rate was suppressed by JuvCon song. The unit on the left showed a consistent increase in firing rate during the first second of song playback, but not at precisely the same time point, as documented in previous studies (Doupe and Solis, 1997; Olveczky et al., 2005; Kao and Brainard, 2006; Kao et al., 2008). Thus, in accord with prior studies in anesthetized birds, firing rates in LMAN neurons are sparse, and song-evoked spikes exhibit a high level of trial-to-trial variability.

**Figure 7.**
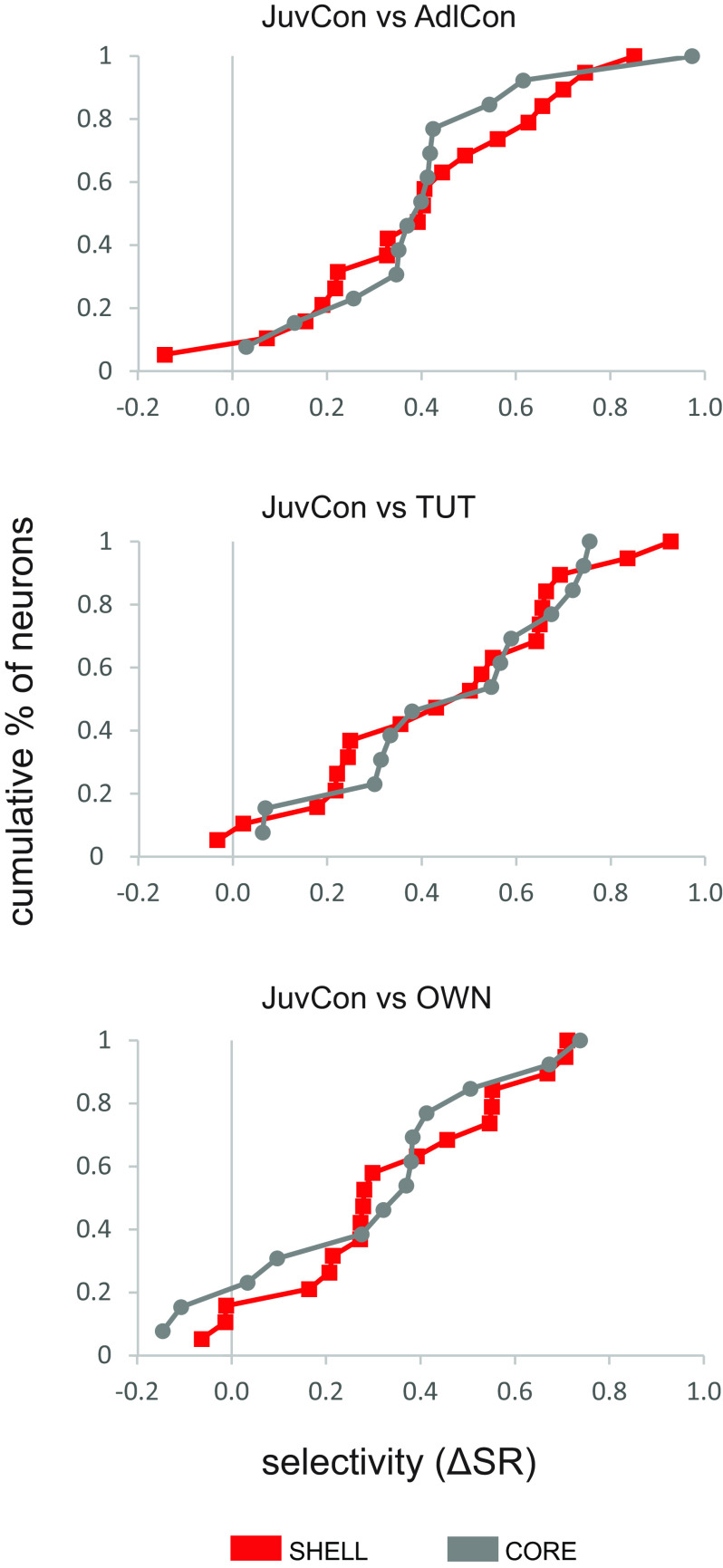
CORE and SHELL neurons were equally selective for JuvCon song. Each panel shows cumulative distribution functions of selectivity scores for JuvCon song compared with AdlCon (top), TUT (middle), and OWN (bottom; *n* = 13 CORE, *n* = 19 SHELL). Positive difference scores indicate a preference for JuvCon song over comparison songs, and show that both CORE (gray) and SHELL (red) neurons preferred JuvCon song over comparison songs to the same extent.

Given the prevalence of suppressed responses to JuvCon songs (Fig. 3; Tables 1, 2), we examined neural selectivity between pairs of stimuli for JuvCon-suppressed neurons by calculating the difference in response strength between song types (see Materials and Methods). Response strengths to OWN, AdlCon, and TUT were subtracted from significantly suppressed JuvCon responses for each cell. A positive difference score indicates that a neuron preferred JuvCon song over comparison songs. Figure 7 shows cumulative distributions of difference scores in CORE versus SHELL neurons for JuvCon against each of the three other song types (*n* = 13 CORE, *n* = 19 SHELL). CORE and SHELL neurons clearly showed the same degree of preference for JuvCon song (Kolmogorov–Smirnov tests, *p* always > 0.87). A similar pattern of selectivity in CORE versus SHELL neurons was obtained when we compared cumulative distributions of difference scores for OWN (*n* = 9 CORE, *n* = 18 SHELL) against each of the three other song types (data not shown). Furthermore, very few neurons exhibited negative selectivity scores; the preponderance of positive scores in Figure 7 shows that cells that exhibited significant suppression to JuvCon almost never showed greater suppression to any other song stimulus. For example, only one SHELL neuron and no CORE neurons showed stronger suppression to AdlCon compared with JuvCon (Fig. 7). One-sample Wilcoxon signed-rank tests to assess whether the distributions were different from zero always yielded *p* values of <0.0001.

## Discussion

The overall pattern of song-evoked responses that we observed in this study contrasted markedly with that in our previous work (Achiro and Bottjer, 2013). Despite the fact that birds of the same age from our breeding colony were used, and all experimental procedures and analyses were the same between the two studies, song-evoked responses in sleeping birds (this study) were substantially different from those observed in our previous work using anesthetized birds (Table 3). Comparing the current results to our prior study, in the present study, the overall level of song-evoked responsivity was lower, responses were overwhelmingly suppressed instead of excited, the incidence of SHELL neurons selectively tuned to tutor song was extremely low, and neurons in both CORE and SHELL tended to show a preference for JuvCon and OWN songs. The preference for juvenile over adult songs is similar to that reported by Yuan and Bottjer (2019) for juvenile RA neurons, which receive direct input from LMAN-CORE, raising the possibility that this preference arises in the corticothalamic circuit that includes CORE.

### Responses in awake versus sleeping or anesthetized states of adult animals

Neural responses to song playback in the motor pathway of the song system, including the cortical regions HVC and RA (Fig. 1), are greatly diminished or absent in awake adult male songbirds but are unmasked under anesthesia or in sleep (Dave et al., 1998; Schmidt and Konishi, 1998; Dave and Margoliash, 2000; Cardin and Schmidt, 2003, 2004a, b; Rauske et al., 2003). This pattern may reflect, at least in part, a general tendency for responses in auditory and/or sensorimotor brain regions to be suppressed during self-generated sounds (Suga and Shimozawa, 1974; Poulet and Hedwig, 2006, 2007; Eliades and Wang, 2008; Singla et al., 2017). For example, neurons in auditory cortex of marmosets show suppression during vocal production; however, responses to their self-generated vocalizations are unmasked when auditory feedback is altered by real-time frequency shifts delivered through headphones (Eliades and Wang, 2008). One idea to arise from such findings is that learned signals from motor or other nonauditory inputs can predict auditory feedback and cancel responses to corresponding auditory sounds. A variant of this idea might explain the absence of song-evoked responses in awake songbirds; for example, motor circuits or pathways for efference copy might act to suppress auditory responses in an awake state even in the absence of active vocalizing. More broadly, the tendency for responses in awake or vocalizing animals to be suppressed is consistent with the idea that behavioral state can regulate a “gate” that controls auditory input.

Qualitative comparisons showing the similarity between song-evoked responses in sleeping versus anesthetized adult male songbirds showed that sleep and anesthesia entail similar behavioral states (Dave et al., 1998; Dave and Margoliash, 2000; Nick and Konishi, 2001). In both anesthetized and sleeping adult songbirds, neurons throughout the song system are selectively tuned to each individual bird’s own song (Margoliash, 1983, 1986; Margoliash and Fortune, 1992; Vicario and Yohay, 1993; Nick and Konishi, 2001; Cardin and Schmidt, 2003; Person and Perkel, 2007). Cardin and Schmidt (2003) directly compared responses of HVC neurons in anesthetized, sleeping/drowsy, and awake adult zebra finches; responses in both anesthetized and sleeping birds were consistently selective for OWN songs, whereas responses in waking birds were highly variable and not selective for OWN. Responses to playback in awake birds reflected the level of arousal: higher levels of arousal uniformly suppressed song-evoked responses in HVC (but had no effect in primary auditory cortex). The similarity of selective responses to OWN song in sleeping and anesthetized birds encouraged the idea that similar behavioral states underlie sleep and anesthesia.

### Responses in awake versus sleeping or anesthetized states of juvenile animals

Very few studies have examined responses to song playback in juvenile songbirds during sleep. Nick and Konishi (2005a) reported that multiunit responses in HVC were strongest to tutor song in awake juvenile zebra finches during early sensorimotor integration, whereas OWN was preferred over tutor songs during sleep. Selectivity for OWN song changed over development in a pattern that tracked the current motor version of each bird’s song (Nick and Konishi, 2005b). Spontaneous patterns of spiking in HVC during sleep also change over song development: both firing rate and bursting increase with age (Crandall et al., 2007).

We are not aware of any previous studies that recorded the response of LMAN neurons to song playback during sleep in juvenile songbirds. Comparison of the present results in juvenile sleeping birds with those of our prior work in anesthetized juveniles (Achiro and Bottjer, 2013) clearly shows that responses of LMAN neurons during sleep are substantially different from those recorded under urethane anesthesia in zebra finches during early sensorimotor integration. Salient differences in LMAN activity between this study and our previously published work are summarized in Table 3. Activity patterns in anesthetized birds differed between CORE and SHELL for each of the measures listed in Table 3, whereas none of these measures varied between regions in sleeping birds. Two particularly striking differences are the dominance of suppressed responses in the present study, and the lack of a prominent neuronal subpopulation that responds selectively to tutor song in SHELL as is seen in anesthetized birds. These differences raise the question of when and how the tutor-tuned SHELL neurons are used in the service of learning. Perhaps our sleep conditions were somehow not conducive to eliciting responses from tutor-selective neurons, in which case they may have an important sleep-related function under other sleep conditions. Or perhaps these neurons are actively involved in some aspect of learning during sleep but are gated off from activation via external auditory playback. Another possibility is that tutor-tuned neurons can be activated during awake states (as for HVC neurons of juvenile birds; Nick and Konishi, 2005a), particularly during singing. If tutor-tuned neurons in awake birds are activated only during singing, their activity might be difficult to identify in the context of motor-related activity (Achiro et al., 2017).

Sleep is essential for vocal learning in juvenile zebra finches (Dave and Margoliash, 2000; Derégnaucourt et al., 2005; Crandall et al., 2007; Shank and Margoliash, 2009; Margoliash and Schmidt, 2010), which brings into question the influence of developmental changes in song-evoked activity during sleep, patterns of spontaneous spiking, and maturation of EEG patterns (see below). Such changes within sensorimotor song regions may be related to substantial changes in the neural substrate for song learning (Alvarez-Buylla et al., 1988; Nordeen and Nordeen, 1988a,b; Herrmann and Arnold, 1991; Johnson and Bottjer, 1992, 1993, 1994; Nordeen et al., 1992; Livingston and Mooney, 1997; Foster and Bottjer, 1998; Iyengar et al., 1999; Kittelberger and Mooney, 1999; Livingston et al., 2000; Nixdorf-Bergweiler, 2001; Iyengar and Bottjer, 2002a, b; Bottjer, 2005; Miller-Sims and Bottjer, 2012; Garst-Orozco et al., 2014; Chung and Bottjer, 2022). For example, axonal projections that are present only during early stages of sensorimotor integration may mediate temporally restricted processes of song learning (Miller-Sims and Bottjer, 2012; Chung and Bottjer, 2022); in addition, refinement of axonal connectivity may represent either a morphologic correlate of song learning or a necessary prerequisite for acquisition of song (Iyengar and Bottjer, 2002a, b). Developmental changes in sleep activity as well as in the neural substrate are likely to be related to changing patterns of responsivity to different song types at different stages of learning. A promising area for investigation lies in the extent to which developmental changes in sleep activity, the underlying neural substrate, selectivity for different song types, and maturation of vocal motor production are interrelated.

### Comparing urethane anesthesia and different sleep states

EEG patterns are not a reliable indicator of sleep in juvenile zebra finches; the amplitude of 1- to 4-Hz activity (δ, an indicator of slow-wave sleep) did not vary between sleep and wake states in zebra finches between 45–65 dph (Nick and Konishi, 2005a; Crandall et al., 2007). The cortical EEG also does not show evidence of state-dependent activity in early postnatal mammals (Gramsbergen, 1976; Frank and Heller, 1997; Blumberg et al., 2005). Even after EEG patterns differentiate (≥12 d postnatal in rodents), a long period of developmental changes ensues, which may be related to maturational changes that facilitate normal development of the nervous system (Khazipov and Luhmann, 2006; Cirelli and Tononi, 2015; Rensing et al., 2018). In any case, these developmental changes complicate efforts to judge similarity between behavioral states in sleep and anesthesia.

Given the similar patterns of song-evoked activity in sleeping and anesthetized adult songbirds, before beginning this study, we assumed that responses to song playback during sleep in LMAN of juvenile birds would replicate our previous results in anesthetized birds. Because we did not intend to study sleep-related factors we made no effort to characterize different stages of sleep in relation to playback. Despite the fact that EEG patterns do not correlate with sleep stages in juvenile animals (Gramsbergen, 1976; Frank and Heller, 1997; Blumberg et al., 2005; Nick and Konishi, 2005a; Crandall et al., 2007; Cirelli and Tononi, 2015), different states of sleep and/or ultradian rhythms may nevertheless influence song responsivity. If so, different sleep states might provide a possible alternative explanation of the stark differences we observed between song-evoked activity in LMAN of sleeping versus anesthetized juvenile zebra finches. Robust responses to song playback are observed during slow wave sleep in HVC of adult zebra finches (Nick and Konishi, 2001). We are not aware of any studies that have compared song-evoked responses during REM (rapid eye movement) versus non-REM sleep. It would be interesting to correlate responsivity to song playback with EEG patterns in adult birds, taking into account that episodes of different sleep states are quite brief (<30 s in adult budgies) and slow wave sleep decreases through the night while REM sleep increases (Canavan and Margoliash, 2020). It is not clear how informative this approach might be in young songbirds given that EEG patterns are not a reliable indicator of sleep states in juvenile animals, although it is nevertheless possible that a given song type could elicit different neural responses in sensorimotor song regions depending on EEG activity.

The similarity of selective responses to OWN songs under sleep and anesthesia in HVC neurons of adult songbirds has encouraged the idea that behavioral states are highly similar between the two conditions. However, this idea has not been extensively tested in either birds or mammals. Some studies have suggested that urethane anesthesia mimics sleep, based on alternation of EEG patterns between a slow-wave state that resembles non-REM sleep and an “activated” state with features of both REM sleep and waking (Clement et al., 2008; Pagliardini et al., 2013; Tisdale et al., 2018; Hauer et al., 2021; Silver et al., 2021). Recent work has not supported this idea, based on detailed comparisons that measured several correlates to define waking versus sleeping states, including power spectra of EEGs, synchronization between high-frequency (γ) oscillations in different brain regions, directional patterns of activation, and temporal complexity of neural oscillations (Mashour and Hudetz, 2018; Kelz and Mashour, 2019; Mashour et al., 2020). Within-subject comparisons of sleep versus urethane anesthesia in rats indicated that these EEG correlates of consciousness were significantly lower during anesthesia compared with sleep (Mondino et al., 2021). For example, normalized power of δ oscillations was higher during both “REM-like” and “non-REM-like” states of urethane anesthesia compared with their respective REM and non-REM states during sleep. These authors concluded that urethane induces a pattern of “sustained unconsciousness” dissimilar from that of sleep. Thus, it seems likely that differences in patterns of brain activity between sleep and anesthesia could underlie the different responses to song playback that we observed in LMAN of juvenile zebra finches between the current study and our previous work (Achiro and Bottjer, 2013). If so, that would suggest that urethane anesthesia is more effective at removing one or more gates of song-evoked activity compared with sleep.

## Synthesis

Reviewing Editor: Silvia Pagliardini, University of Alberta Decisions are customarily a result of the Reviewing Editor and the peer reviewers coming together and discussing their recommendations until a consensus is reached. When revisions are invited, a fact-based synthesis statement explaining their decision and outlining what is needed to prepare a revision will be listed below. The following reviewer(s) agreed to reveal their identity: NONE.

The authors have addressed all of the concerns raised during the initial manuscript review.

## References

[B1] Achiro JM, Bottjer SW (2013) Neural representation of a target auditory memory in a cortico-basal ganglia pathway. J Neurosci 33:14475–14488. 10.1523/JNEUROSCI.0710-13.2013 24005299 PMC3761053

[B2] Achiro JM, Shen J, Bottjer SW (2017) Neural activity in cortico-basal ganglia circuits of juvenile songbirds encodes performance during goal-directed learning. Elife 6:e26973. 10.7554/eLife.26973 29256393 PMC5762157

[B3] Adret P, Meliza CD, Margoliash D (2012) Song tutoring in presinging zebra finch juveniles biases a small population of higher-order song-selective neurons toward the tutor song. J Neurophysiol 108:1977–1987. 10.1152/jn.00905.2011 22786956 PMC3544995

[B4] Alexander GE, Crutcher MD (1990) Functional architecture of basal ganglia circuits: neural substrates of parallel processing. Trends Neurosci 13:266–271. 10.1016/0166-2236(90)90107-l 1695401

[B5] Alvarez-Buylla A, Theelen M, Nottebohm F (1988) Birth of projection neurons in the higher vocal center of the canary forebrain before, during and after song learning. Proc Natl Acad Sci U S A 85:8722–8726. 10.1073/pnas.85.22.8722 3186755 PMC282533

[B6] Aronov D, Andalman AS, Fee MS (2008) A specialized forebrain circuit for vocal babbling in the juvenile songbird. Science 320:630–634. 10.1126/science.1155140 18451295

[B7] Ashby FG, Turner BO, Horvitz JC (2010) Cortical and basal ganglia contributions to habit learning and automaticity. Trends Cogn Sci 14:208–215. 10.1016/j.tics.2010.02.001 20207189 PMC2862890

[B8] Benjamini Y, Hochberg Y (1995) Controlling the false discovery rate - a practical and powerful approach to multiple testing. J Roy Stat Soc B Met 57:289–300. 10.1111/j.2517-6161.1995.tb02031.x

[B9] Blumberg MS, Karlsson KA, Seelke AM, Mohns EJ (2005) The ontogeny of mammalian sleep: a response to Frank and Heller (2003). J Sleep Res 14:91–98. 10.1111/j.1365-2869.2004.00430_1.x 15743339 PMC2637352

[B10] Böhner J (1983) Song learning in the zebra finch (*Taeniopygia guttata*): selectivity in the choice of a tutor and accuracy of song copies. Anim Behav 31:231–237. 10.1016/S0003-3472(83)80193-6

[B11] Böhner J (1990) Early acquisition of song in the zebra finch, *Taeniopygia guttata*. Anim Behav 39:369–374. 10.1016/S0003-3472(05)80883-8

[B12] Bottjer SW (2004) Developmental regulation of basal ganglia circuitry during the sensitive period for vocal learning in songbirds. Ann N Y Acad Sci 1016:395–415. 10.1196/annals.1298.037 15313787

[B13] Bottjer SW (2005) Silent synapses in a thalamo-cortical circuit necessary for song learning in zebra finches. J Neurophysiol 94:3698–3707. 10.1152/jn.00282.2005 16107531

[B14] Bottjer SW, Altenau B (2010) Parallel pathways for vocal learning in basal ganglia of songbirds. Nat Neurosci 13:153–155. 10.1038/nn.2472 20023650 PMC2846604

[B15] Bottjer SW, Miesner EA, Arnold AP (1984) Forebrain lesions disrupt development but not maintenance of song in passerine birds. Science 224:901–903. 10.1126/science.6719123 6719123

[B16] Brainard MS, Doupe AJ (2013) Translating birdsong: songbirds as a model for basic and applied medical research. Annu Rev Neurosci 36:489–517. 10.1146/annurev-neuro-060909-152826 23750515 PMC4130661

[B17] Canavan SV, Margoliash D (2020) Budgerigars have complex sleep structure similar to that of mammals. PLoS Biol 18:e3000929. 10.1371/journal.pbio.3000929 33201883 PMC7707536

[B18] Cardin JA, Schmidt MF (2003) Song system auditory responses are stable and highly tuned during sedation, rapidly modulated and unselective during wakefulness, and suppressed by arousal. J Neurophysiol 90:2884–2899. 10.1152/jn.00391.2003 12878713

[B19] Cardin JA, Schmidt MF (2004a) Auditory responses in multiple sensorimotor song system nuclei are co-modulated by behavioral state. J Neurophysiol 91:2148–2163. 10.1152/jn.00918.2003 14724261

[B20] Cardin JA, Schmidt MF (2004b) Noradrenergic inputs mediate state dependence of auditory responses in the avian song system. J Neurosci 24:7745–7753. 10.1523/JNEUROSCI.1951-04.2004 15342742 PMC6729633

[B21] Chung JH, Bottjer SW (2022) Developmentally regulated pathways for motor skill learning in songbirds. J Comp Neurol 530:1288–1301. 34818442 10.1002/cne.25276 PMC8969184

[B22] Cirelli C, Tononi G (2015) Cortical development, electroencephalogram rhythms, and the sleep/wake cycle. Biol Psychiatry 77:1071–1078. 10.1016/j.biopsych.2014.12.017 25680672 PMC4444390

[B23] Clement EA, Richard A, Thwaites M, Ailon J, Peters S, Dickson CT (2008) Cyclic and sleep-like spontaneous alternations of brain state under urethane anaesthesia. PLoS One 3:e2004. 10.1371/journal.pone.0002004 18414674 PMC2289875

[B24] Coleman MJ, Mooney R (2004) Synaptic transformations underlying highly selective auditory representations of learned birdsong. J Neurosci 24:7251–7265. 10.1523/JNEUROSCI.0947-04.2004 15317851 PMC6729779

[B25] Cox J, Witten IB (2019) Striatal circuits for reward learning and decision-making. Nat Rev Neurosci 20:482–494. 10.1038/s41583-019-0189-2 31171839 PMC7231228

[B26] Crandall SR, Adam M, Kinnischtzke AK, Nick TA (2007) HVC neural sleep activity increases with development and parallels nightly changes in song behavior. J Neurophysiol 98:232–240. 10.1152/jn.00128.2007 17428907 PMC2268767

[B27] Dave AS, Margoliash D (2000) Song replay during sleep and computational rules for sensorimotor vocal learning. Science 290:812–816. 10.1126/science.290.5492.812 11052946

[B28] Dave AS, Yu AC, Margoliash D (1998) Behavioral state modulation of auditory activity in a vocal motor system. Science 282:2250–2254. 10.1126/science.282.5397.2250 9856946

[B29] Derégnaucourt S, Mitra PP, Feher O, Pytte C, Tchernichovski O (2005) How sleep affects the developmental learning of bird song. Nature 433:710–716. 10.1038/nature03275 15716944

[B30] Doupe AJ (1997) Song- and order-selective neurons in the songbird anterior forebrain and their emergence during vocal development. J Neurosci 17:1147–1167. 10.1523/JNEUROSCI.17-03-01147.1997 8994068 PMC6573158

[B31] Doupe AJ, Solis MM (1997) Song- and order-selective neurons develop in the songbird anterior forebrain during vocal learning. J Neurobiol 33:694–709. 10.1002/(SICI)1097-4695(19971105)33:5<694::AID-NEU13>3.0.CO;2-9 9369467

[B32] Doupe AJ, Kuhl PK (1999) Birdsong and human speech: common themes and mechanisms. Annu Rev Neurosci 22:567–631. 10.1146/annurev.neuro.22.1.567 10202549

[B33] Eliades SJ, Wang X (2008) Neural substrates of vocalization feedback monitoring in primate auditory cortex. Nature 453:1102–1106. 10.1038/nature06910 18454135

[B34] Elliott KC, Wu W, Bertram R, Johnson F (2014) Disconnection of a basal ganglia circuit in juvenile songbirds attenuates the spectral differentiation of song syllables. Dev Neurobiol 74:574–590. 10.1002/dneu.22151 24218118 PMC4120835

[B35] Foster EF, Bottjer SW (1998) Axonal connections of the high vocal center and surrounding cortical regions in juvenile and adult male zebra finches. J Comp Neurol 397:118–138. 10.1002/(SICI)1096-9861(19980720)397:1<118::AID-CNE9>3.0.CO;2-3 9671283

[B36] Frank MG, Heller HC (1997) Development of REM and slow wave sleep in the rat. Am J Physiol 272:R1792–R1799. 10.1152/ajpregu.1997.272.6.R1792 9227592

[B37] Gale SD, Person AL, Perkel DJ (2008) A novel basal ganglia pathway forms a loop linking a vocal learning circuit with its dopaminergic input. J Comp Neurol 508:824–839. 10.1002/cne.21700 18398824

[B38] Garst-Orozco J, Babadi B, Ölveczky BP (2014) A neural circuit mechanism for regulating vocal variability during song learning in zebra finches. Elife 3:e03697. 10.7554/eLife.03697 25497835 PMC4290448

[B39] Gramsbergen A (1976) The development of the EEG in the rat. Dev Psychobiol 9:501–515. 10.1002/dev.420090604 1001836

[B40] Graybiel AM (2008) Habits, rituals, and the evaluative brain. Annu Rev Neurosci 31:359–387. 10.1146/annurev.neuro.29.051605.112851 18558860

[B41] Gremel CM, Costa RM (2013) Orbitofrontal and striatal circuits dynamically encode the shift between goal-directed and habitual actions. Nat Commun 4:2264. 10.1038/ncomms3264 23921250 PMC4026062

[B42] Hahnloser RH, Kozhevnikov AA, Fee MS (2002) An ultra-sparse code underlies the generation of neural sequences in a songbird. Nature 419:65–70. 10.1038/nature00974 12214232

[B43] Hahnloser RH, Wang CZ, Nager A, Naie K (2008) Spikes and bursts in two types of thalamic projection neurons differentially shape sleep patterns and auditory responses in a songbird. J Neurosci 28:5040–5052. 10.1523/JNEUROSCI.5059-07.2008 18463257 PMC6670741

[B44] Hauer BE, Pagliardini S, Dickson CT (2021) Prefrontal-hippocampal pathways through the nucleus reuniens are functionally biased by brain state. Front Neuroanat 15:804872. 10.3389/fnana.2021.804872 35173588 PMC8842257

[B45] Herrmann K, Arnold AP (1991) The development of afferent projections to the robust archistriatal nucleus in male zebra finches: a quantitative electron microscopic study. J Neurosci 11:2063–2074. 10.1523/JNEUROSCI.11-07-02063.1991 2066775 PMC6575480

[B46] Iyengar S, Bottjer SW (2002a) Development of individual axon arbors in a thalamocortical circuit necessary for song learning in zebra finches. J Neurosci 22:901–911. 10.1523/JNEUROSCI.22-03-00901.2002 11826119 PMC6758476

[B47] Iyengar S, Bottjer SW (2002b) The role of auditory experience in the formation of neural circuits underlying vocal learning in zebra finches. J Neurosci 22:946–958. 10.1523/JNEUROSCI.22-03-00946.2002 11826123 PMC6758492

[B48] Iyengar S, Viswanathan SS, Bottjer SW (1999) Development of topography within song control circuitry of zebra finches during the sensitive period for song learning. J Neurosci 19:6037–6057. 10.1523/JNEUROSCI.19-14-06037.1999 10407041 PMC6783091

[B49] Johnson F, Bottjer SW (1992) Growth and regression of thalamic efferents in the song-control system of male zebra finches. J Comp Neurol 326:442–450. 10.1002/cne.903260309 1469121

[B50] Johnson F, Bottjer SW (1993) Induced cell death in a thalamic nucleus during a restricted period of zebra finch vocal development. J Neurosci 13:2452–2462. 10.1523/JNEUROSCI.13-06-02452.1993 8501517 PMC6576511

[B51] Johnson F, Bottjer SW (1994) Afferent influences on cell death and birth during development of a cortical nucleus necessary for learned vocal behavior in zebra finches. Development 120:13–24. 10.1242/dev.120.1.13 21375056

[B52] Johnson F, Sablan MM, Bottjer SW (1995) Topographic organization of a forebrain pathway involved with vocal learning in zebra finches. J Comp Neurol 358:260–278. 10.1002/cne.903580208 7560286

[B53] Kao MH, Brainard MS (2006) Lesions of an avian basal ganglia circuit prevent context-dependent changes to song variability. J Neurophysiol 96:1441–1455. 10.1152/jn.01138.2005 16723412

[B54] Kao MH, Wright BD, Doupe AJ (2008) Neurons in a forebrain nucleus required for vocal plasticity rapidly switch between precise firing and variable bursting depending on social context. J Neurosci 28:13232–13247. 10.1523/JNEUROSCI.2250-08.2008 19052215 PMC3022006

[B55] Kelz MB, Mashour GA (2019) The biology of general anesthesia from paramecium to primate. Curr Biol 29:R1199–R1210. 10.1016/j.cub.2019.09.071 31743680 PMC6902878

[B56] Khazipov R, Luhmann HJ (2006) Early patterns of electrical activity in the developing cerebral cortex of humans and rodents. Trends Neurosci 29:414–418. 10.1016/j.tins.2006.05.007 16713634

[B57] Kittelberger JM, Mooney R (1999) Lesions of an avian forebrain nucleus that disrupt song development alter synaptic connectivity and transmission in the vocal premotor pathway. J Neurosci 19:9385–9398. 10.1523/JNEUROSCI.19-21-09385.1999 10531443 PMC6782913

[B58] Kojima S, Doupe AJ (2007) Song selectivity in the pallial-basal ganglia song circuit of zebra finches raised without tutor song exposure. J Neurophysiol 98:2099–2109. 10.1152/jn.00916.2006 17625059 PMC2443789

[B59] Kojima S, Kao MH, Doupe AJ, Brainard MS (2018) The avian basal ganglia are a source of rapid behavioral variation that enables vocal motor exploration. J Neurosci 38:9635–9647. 10.1523/JNEUROSCI.2915-17.2018 30249800 PMC6222063

[B60] Kupferschmidt DA, Juczewski K, Cui G, Johnson KA, Lovinger DM (2017) Parallel, but dissociable, processing in discrete corticostriatal inputs encodes skill learning. Neuron 96:476–489.e5. 10.1016/j.neuron.2017.09.040 29024667 PMC5663197

[B61] Lewicki MS, Konishi M (1995) Mechanisms underlying the sensitivity of songbird forebrain neurons to temporal order. Proc Natl Acad Sci U S A 92:5582–5586. 10.1073/pnas.92.12.5582 7777552 PMC41740

[B62] Livingston FS, Mooney R (1997) Development of intrinsic and synaptic properties in a forebrain nucleus essential to avian song learning. J Neurosci 17:8997–9009. 10.1523/JNEUROSCI.17-23-08997.1997 9364047 PMC6573603

[B63] Livingston FS, White SA, Mooney R (2000) Slow NMDA-EPSCs at synapses critical for song development are not required for song learning in zebra finches. Nat Neurosci 3:482–488. 10.1038/74857 10769389

[B64] Low PS, Shank SS, Sejnowski TJ, Margoliash D (2008) Mammalian-like features of sleep structure in zebra finches. Proc Natl Acad Sci U S A 105:9081–9086. 10.1073/pnas.0703452105 18579776 PMC2440357

[B65] Ludwig KA, Miriani RM, Langhals NB, Joseph MD, Anderson DJ, Kipke DR (2009) Using a common average reference to improve cortical neuron recordings from microelectrode arrays. J Neurophysiol 101:1679–1689. 10.1152/jn.90989.2008 19109453 PMC2666412

[B66] Luo M, Ding L, Perkel DJ (2001) An avian basal ganglia pathway essential for vocal learning forms a closed topographic loop. J Neurosci 21:6836–6845. 10.1523/JNEUROSCI.21-17-06836.2001 11517271 PMC6763103

[B67] Mann NI, Slater PJB (1995) Song tutor choice by zebra finches in aviaries. Anim Behav 49:811–820. 10.1016/0003-3472(95)90054-3 10053084

[B68] Mann NI, Slater PJB, Eales LA, Richards C (1991) The influence of visual stimuli on song tutor choice in the zebra finch, *Taenopygia guttata*. Anim Behav 42:285–293. 10.1016/S0003-3472(05)80560-3

[B69] Margoliash D (1983) Acoustic parameters underlying the responses of song-specific neurons in the white-crowned sparrow. J Neurosci 3:1039–1057. 10.1523/JNEUROSCI.03-05-01039.1983 6842281 PMC6564505

[B70] Margoliash D (1986) Preference for autogenous song by auditory neurons in a song system nucleus of the white-crowned sparrow. J Neurosci 6:1643–1661. 10.1523/JNEUROSCI.06-06-01643.1986 3712002 PMC6568716

[B71] Margoliash D, Konishi M (1985) Auditory representation of autogenous song in the song system of white-crowned sparrows. Proc Natl Acad Sci U S A 82:5997–6000. 10.1073/pnas.82.17.5997 16593601 PMC390681

[B72] Margoliash D, Fortune ES (1992) Temporal and harmonic combination-sensitive neurons in the zebra finch’s HVc. J Neurosci 12:4309–4326. 10.1523/JNEUROSCI.12-11-04309.1992 1432096 PMC6575994

[B73] Margoliash D, Schmidt MF (2010) Sleep, off-line processing, and vocal learning. Brain Lang 115:45–58. 10.1016/j.bandl.2009.09.005 19906416 PMC2891378

[B74] Mashour GA, Hudetz AG (2018) Neural correlates of unconsciousness in large-scale brain networks. Trends Neurosci 41:150–160. 10.1016/j.tins.2018.01.003 29409683 PMC5835202

[B75] Mashour GA, Roelfsema P, Changeux JP, Dehaene S (2020) Conscious processing and the global neuronal workspace hypothesis. Neuron 105:776–798. 10.1016/j.neuron.2020.01.026 32135090 PMC8770991

[B76] Miller-Sims VC, Bottjer SW (2012) Auditory experience refines cortico-basal ganglia inputs to motor cortex via remapping of single axons during vocal learning in zebra finches. J Neurophysiol 107:1142–1156. 10.1152/jn.00614.2011 22157116 PMC3289456

[B77] Mondino A, González J, Li D, Mateos D, Osorio L, Cavelli M, Costa A, Vanini G, Mashour G, Torterolo P (2021) Urethane anesthesia exhibits neurophysiological correlates of unconsciousness and is distinct from sleep. bioRxiv. doi: 10.1101/2021.09.21.461281 35545450

[B78] Nick TA, Konishi M (2001) Dynamic control of auditory activity during sleep: correlation between song response and EEG. Proc Natl Acad Sci U S A 98:14012–14016. 10.1073/pnas.251525298 11717459 PMC61158

[B79] Nick TA, Konishi M (2005a) Neural song preference during vocal learning in the zebra finch depends on age and state. J Neurobiol 62:231–242. 10.1002/neu.20087 15459895

[B80] Nick TA, Konishi M (2005b) Neural auditory selectivity develops in parallel with song. J Neurobiol 62:469–481. 10.1002/neu.20115 15616963

[B81] Nixdorf-Bergweiler BE (2001) Lateral magnocellular nucleus of the anterior neostriatum (LMAN) in the zebra finch: neuronal connectivity and the emergence of sex differences in cell morphology. Microsc Res Tech 54:335–353. 10.1002/jemt.1147 11668647

[B82] Nordeen EJ, Nordeen KW (1988a) Sex and regional differences in the incorporation of neurons born during song learning in zebra finches. J Neurosci 8:2869–2874. 10.1523/JNEUROSCI.08-08-02869.1988 3411358 PMC6569406

[B84] Nordeen KW, Nordeen EJ (1988b) Projection neurons within a vocal motor pathway are born during song learning in zebra finches. Nature 334:149–151. 10.1038/334149a0 3386754

[B83] Nordeen EJ, Grace A, Burek MJ, Nordeen KW (1992) Sex-dependent loss of projection neurons involved in avian song learning. J Neurobiol 23:671–679. 10.1002/neu.480230606 1279116

[B85] Olveczky BP, Andalman AS, Fee MS (2005) Vocal experimentation in the juvenile songbird requires a basal ganglia circuit. PLoS Biol 3:e153. 10.1371/journal.pbio.0030153 15826219 PMC1069649

[B86] Pagliardini S, Gosgnach S, Dickson CT (2013) Spontaneous sleep-like brain state alternations and breathing characteristics in urethane anesthetized mice. PLoS One 8:e70411. 10.1371/journal.pone.0070411 23936201 PMC3728022

[B87] Paterson AK, Bottjer SW (2017) Cortical inter-hemispheric circuits for multimodal vocal learning in songbirds. J Comp Neurol 525:3312–3340. 10.1002/cne.24280 28681379 PMC6301027

[B88] Person AL, Perkel DJ (2007) Pallidal neuron activity increases during sensory relay through thalamus in a songbird circuit essential for learning. J Neurosci 27:8687–8698. 10.1523/JNEUROSCI.2045-07.2007 17687046 PMC6672941

[B89] Person AL, Gale SD, Farries MA, Perkel DJ (2008) Organization of the songbird basal ganglia, including area X. J Comp Neurol 508:840–866. 10.1002/cne.21699 18398825

[B90] Poulet JF, Hedwig B (2006) The cellular basis of a corollary discharge. Science 311:518–522. 10.1126/science.1120847 16439660

[B91] Poulet JF, Hedwig B (2007) New insights into corollary discharges mediated by identified neural pathways. Trends Neurosci 30:14–21. 10.1016/j.tins.2006.11.005 17137642

[B92] Rauske PL, Shea SD, Margoliash D (2003) State and neuronal class-dependent reconfiguration in the avian song system. J Neurophysiol 89:1688–1701. 10.1152/jn.00655.2002 12626633

[B93] Redgrave P, Rodriguez M, Smith Y, Rodriguez-Oroz MC, Lehericy S, Bergman H, Agid Y, DeLong MR, Obeso JA (2010) Goal-directed and habitual control in the basal ganglia: implications for Parkinson’s disease. Nat Rev Neurosci 11:760–772. 10.1038/nrn2915 20944662 PMC3124757

[B94] Rensing N, Moy B, Friedman JL, Galindo R, Wong M (2018) Longitudinal analysis of developmental changes in electroencephalography patterns and sleep-wake states of the neonatal mouse. PLoS One 13:e0207031. 10.1371/journal.pone.0207031 30399187 PMC6219806

[B95] Roper A, Zann R (2006) The onset of song learning and song tutor selection in fledgling zebra finches. Ethology 112:458–470. 10.1111/j.1439-0310.2005.01169.x

[B96] Scharff C, Nottebohm F (1991) A comparative study of the behavioral deficits following lesions of various parts of the zebra finch song system: implications for vocal learning. J Neurosci 11:2896–2913. 1880555 10.1523/JNEUROSCI.11-09-02896.1991 PMC6575264

[B97] Schmidt MF, Konishi M (1998) Gating of auditory responses in the vocal control system of awake songbirds. Nat Neurosci 1:513–518. 10.1038/2232 10196550

[B98] Shank SS, Margoliash D (2009) Sleep and sensorimotor integration during early vocal learning in a songbird. Nature 458:73–77. 10.1038/nature07615 19079238 PMC2651989

[B99] Silver NRG, Ward-Flanagan R, Dickson CT (2021) Long-term stability of physiological signals within fluctuations of brain state under urethane anesthesia. PLoS One 16:e0258939. 10.1371/journal.pone.0258939 34695166 PMC8544839

[B100] Singla S, Dempsey C, Warren R, Enikolopov AG, Sawtell NB (2017) A cerebellum-like circuit in the auditory system cancels responses to self-generated sounds. Nat Neurosci 20:943–950. 10.1038/nn.4567 28530663 PMC5525154

[B101] Solis MM, Doupe AJ (1997) Anterior forebrain neurons develop selectivity by an intermediate stage of birdsong learning. J Neurosci 17:6447–6462. 9236252 10.1523/JNEUROSCI.17-16-06447.1997 PMC6568360

[B102] Solis MM, Doupe AJ (1999) Contributions of tutor and bird’s own song experience to neural selectivity in the songbird anterior forebrain. J Neurosci 19:4559–4584. 10.1523/JNEUROSCI.19-11-04559.1999 10341255 PMC6782615

[B103] Solis MM, Doupe AJ (2000) Compromised neural selectivity for song in birds with impaired sensorimotor learning. Neuron 25:109–121. 10.1016/S0896-6273(00)80875-2 10707976

[B104] Steriade M, Llinás RR (1988) The functional states of the thalamus and the associated neuronal interplay. Physiol Rev 68:649–742. 10.1152/physrev.1988.68.3.649 2839857

[B105] Suga N, Shimozawa T (1974) Site of neural attenuation of responses to self-vocalized sounds in echolocating bats. Science 183:1211–1213. 10.1126/science.183.4130.1211 4812353

[B106] Swadlow HA, Gusev AG (2001) The impact of ‘bursting’ thalamic impulses at a neocortical synapse. Nat Neurosci 4:402–408. 10.1038/86054 11276231

[B107] Szymczak JT, Kaiser W, Helb HW, Beszczyńska B (1996) A study of sleep in the European blackbird. Physiol Behav 60:1115–1120. 10.1016/0031-9384(96)00231-4 8884941

[B108] Thorn CA, Atallah H, Howe M, Graybiel AM (2010) Differential dynamics of activity changes in dorsolateral and dorsomedial striatal loops during learning. Neuron 66:781–795. 10.1016/j.neuron.2010.04.036 20547134 PMC3108575

[B109] Tisdale RK, Tieri L, Rattenborg NC, Beckers GJL, Lesku JA (2018) Spectral properties of brain activity under two anesthetics and their potential for inducing natural sleep in birds. Front Neurosci 12:881. 30538619 10.3389/fnins.2018.00881 PMC6277676

[B110] Turner RS, Desmurget M (2010) Basal ganglia contributions to motor control: a vigorous tutor. Curr Opin Neurobiol 20:704–716. 10.1016/j.conb.2010.08.022 20850966 PMC3025075

[B111] Vicario DS, Yohay KH (1993) Song-selective auditory input to a forebrain vocal control nucleus in the zebra finch. J Neurobiol 24:488–505. 10.1002/neu.480240407 8515252

[B112] Volman SF (1993) Development of neural selectivity for birdsong during vocal learning. J Neurosci 13:4737–4747. 8229196 10.1523/JNEUROSCI.13-11-04737.1993 PMC6576335

[B113] Weyand TG, Boudreaux M, Guido W (2001) Burst and tonic response modes in thalamic neurons during sleep and wakefulness. J Neurophysiol 85:1107–1118. 10.1152/jn.2001.85.3.1107 11247981

[B114] Yin HH, Knowlton BJ (2006) The role of the basal ganglia in habit formation. Nat Rev Neurosci 7:464–476. 10.1038/nrn1919 16715055

[B115] Yin HH, Mulcare SP, Hilário MR, Clouse E, Holloway T, Davis MI, Hansson AC, Lovinger DM, Costa RM (2009) Dynamic reorganization of striatal circuits during the acquisition and consolidation of a skill. Nat Neurosci 12:333–341. 10.1038/nn.2261 19198605 PMC2774785

[B116] Yu AC, Margoliash D (1996) Temporal hierarchical control of singing in birds. Science 273:1871–1875. 10.1126/science.273.5283.1871 8791594

[B117] Yuan RC, Bottjer SW (2019) Differential developmental changes in cortical representations of auditory-vocal stimuli in songbirds. J Neurophysiol 121:530–548. 10.1152/jn.00714.2018 30540540 PMC6397395

[B118] Zevin JD, Seidenberg MS, Bottjer SW (2004) Limits on reacquisition of song in adult zebra finches exposed to white noise. J Neurosci 24:5849–5862. 10.1523/JNEUROSCI.1891-04.2004 15229232 PMC6729238

